# Decadal shifts of East Asian summer monsoon in a climate model free of explicit GHGs and aerosols

**DOI:** 10.1038/srep38546

**Published:** 2016-12-09

**Authors:** Renping Lin, Jiang Zhu, Fei Zheng

**Affiliations:** 1 International Center for Climate and Environment Sciences, Institute of Atmospheric Physics, Chinese Academy of Sciences, Beijing 100029, China.

## Abstract

The East Asian summer monsoon (EASM) experienced decadal transitions over the past few decades, and the associated "wetter-South-drier-North" shifts in rainfall patterns in China significantly affected the social and economic development in China. Two viewpoints stand out to explain these decadal shifts, regarding the shifts either a result of internal variability of climate system or that of external forcings (e.g. greenhouse gases (GHGs) and anthropogenic aerosols). However, most climate models, for example, the Atmospheric Model Intercomparison Project (AMIP)-type simulations and the Coupled Model Intercomparison Project (CMIP)-type simulations, fail to simulate the variation patterns, leaving the mechanisms responsible for these shifts still open to dispute. In this study, we conducted a successful simulation of these decadal transitions in a coupled model where we applied ocean data assimilation in the model free of explicit aerosols and GHGs forcing. The associated decadal shifts of the three-dimensional spatial structure in the 1990s, including the eastward retreat, the northward shift of the western Pacific subtropical high (WPSH), and the south-cool-north-warm pattern of the upper-level tropospheric temperature, were all well captured. Our simulation supports the argument that the variations of the oceanic fields are the dominant factor responsible for the EASM decadal transitions.

The East Asian summer monsoon (EASM) is a crucial climate system in Indo-China, the Philippines, China, the Korean Peninsula and Japan^1^. The monsoonal rain occurs in a concentrated belt that stretches east-west during the prevalence of the summer monsoon, accounting for 40–50% (60–70%) of the annual precipitation in South (North) China^2^. Any process that disturbs the normal monsoon rain belt would lead to relative drought or flood throughout East Asia, and affect the economy and society of the region^3^. The EASM underwent a substantial decadal weakening in the late 1970s and then made a strong comeback in the early 1990s^4^,^5^,^6^,^7^. As shown in Fig. 1a, the 7-year low-pass filtered low-level south wind averaged over 110°−120°E exhibits the decadal weakening and shifts southward around the late 1970s, and the decadal strengthening and shifts back northward after the early 1990s. Since the late 1970s, the anomalous summer rainfall in East China shifted to a “wetter-south-drier-north” pattern with the anomalous wetter belt located over the middle to lower reaches of Yangtze River Valley^6^. Later since the early 1990s, with the strengthening and northward shifts of the meridional wind, the anomalous wetter rain belt moves northward, leading to more extreme floods in Northeast China and a dryer Yangtze River in its middle and lower reaches^4^.

From the internal variability view of point, slow variations in a coupled atmosphere-ocean system tend to be controlled by the slowly evolving ocean field, thus the intrinsic oscillation in the ocean is usually regarded as a dominant cause for the decadal variations of the EASM. Observational evidences showed that in the positive (negative) Pacific Decadal Oscillation (PDO) phase, with the warming (cooling) in the central and eastern tropical Pacific, summer precipitation in North China decreases (increases)^8^,^9^. AMIP-type numerical experiments implied that the recent warming over the tropical oceans, especially those associated with the PDO centered over the central and eastern Pacific, has played a major role in the weakening of the EASM during recent decades^10^. Numerical experiments further demonstrated that the shift of PDO from positive to negative phase probably induces warming of the Lake Baikal and the weakening of the westerly jet through the air-sea interaction in the Pacific, changing the summer precipitation pattern in East China^11^,^12^. Some studies argue that, apart from PDO, Atlantic multi-decadal oscillation (AMO) can also modulate the decadal variation of the EASM through the air-sea coupled processes in the Indian Ocean and North Pacific^13^. Meanwhile, others indicate that AMO can influence Mei-yu precipitation and tropospheric temperature of East Asia via a Eurasian wave train emanating from North Atlantic to China^14^. These mechanisms, whether related to PDO or AMO, imply that SST field is a key factor to influence the decadal variation of the EASM, and that unbiased SST may be extremely important for EASM simulation. However, the AMIP-type simulations, in which the atmospheric model is forced by prescribed real SST, have difficulty in reproducing the observed decadal variations of EASM^15^.

With respect to external forcings, some suggest that greenhouse gases (GHGs)-induce global warming, especially the Indian Ocean and western Pacific warm pool warming, may lead to monsoonal precipitation increase in East Asia^16^. Others argue that the slower warming rate in China compared to the surrounding regions may reduce the land-sea thermal contrast in East Asia, which in turn will further weaken the EASM^17^. Besides GHGs, some studies suggested that the anthropogenic aerosols can also contribute to the “wetter-south-dryer-north” phenomenon in East China by heating the air and altering regional atmospheric stability and vertical motions^18^. However, some subsequent studies stated that their model with aerosols cannot produce the “wetter-south-dryer-north” rainfall shift, which may be associated with the totally different estimation of aerosols (e.g., absorbing BC) among independent studies^19^,^20^. Based on the latest CMIP5 models, the relative contributions of GHGs, aerosol, and natural forcing on the EASM were compared^21^. It is found that GHG enhances the EASM while aerosol decreases the EASM. Moreover, the contributions of GHGs and aerosols may be relatively small compared to the internal variability. Also note that the large uncertainties of aerosols parameterizations in models may lead to inconsistency of conclusions.

A realistic simulation of the EASM decadal change is indispensable to enhance our understanding. In this study, we developed a weakly-coupled data assimilation system in which ocean observations are employed to constrain ocean fields of a coupled climate model (CAS-ESM-C^22^; Methods) through a global ocean data assimilation scheme (Methods). The basic behavior of this weakly-coupled data assimilation system has been evaluated on the climatology and interannual variability of the global climate system^23^. By applying ocean data assimilation in CAS-ESM-C, we try to input the internal variability of climate system into the model without breaking the air-sea interaction, and expect to reasonably reproduce the observed decadal variations of the EASM. To our knowledge, this is the first time that the decadal variations of the EASM are well captured in a coupled climate model without GHGs/aerosols forcing.

## Results

**Figure 1:**
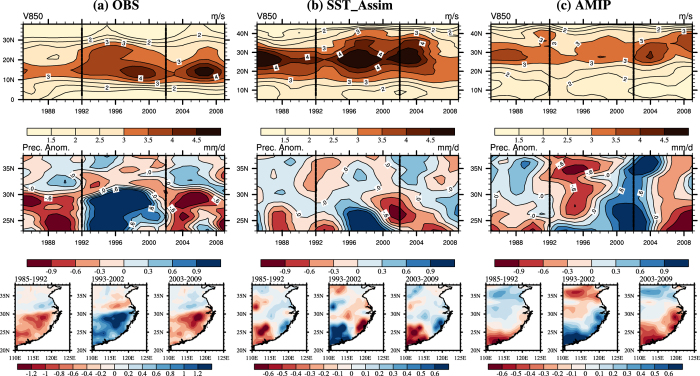
Decadal variation of EASM. The time-latitude distribution of 7-year low-pass filtered meridional wind at 850 hPa (V850) and anomalous rainfall derive from (**a**) OBS, (**b**) SST_Assim and (**c**) AMIP. And the spatial pattern of decadal mean rainfall anomaly averaged over three decadal periods respectively. The V850 and rainfall anomalies are averaged over East China (110°–120°E). The rainfall anomalies are calculated from removing the climatology of 1985 to 2004 and its long term trend. The dashed lines indicate the time of decadal change. The observed V850 and precipitation are derived from NCEP/NCAR reanalysis data and a high resolution gridded precipitation data across China. Maps were generated using NCAR Command Language (The NCAR Command Language (Version 6.1.2) [Software]. (2013). Boulder, Colorado: UCAR/NCAR/CISL/TDD. http://www.ncl.ucar.edu/).

Figure 1b-c shows the decadal variations of the EASM simulated by SST_Assim and AMIP (Methods). Since the time period of the observed daily SST^24^ assimilated into CAS-ESM-C spans from 1981 to 2014, we can only focus on the decadal changes after the middle 1980s here. Main anomalous meridional wind features at 850-hPa (V850) in the observation are well captured by SST_Assim (Fig. 1b), that is the enhancing and northward shift of low-level southerly wind since the early 1990s. Although the simulated southward shifts of V850 since 2006 in SST_Assim is absent in the observation, we can still regard SST_Assim run as more reasonable than AMIP in reproducing the decadal variation of V850.

It is a more complex situation turning to the simulation of precipitation (Fig. 1b-c). From the observation, in the first period (before 1992) the precipitation anomaly shows a north-wetter-south-drier pattern. Both SST_Assim and AMIP run can reproduce this pattern. In the second period (1993–2002), observation shows a north-drier-south-wetter pattern. With respect to the simulation, both SST_Assim and AMIP have large bias in this period. In SST_Assim run, the positive precipitation center in the south extended too far to the north, while in AMIP run, the positive anomaly shifts southward and almost all the region is negative anomaly. Although large bias exists, it still should be noted that the positive precipitation center in the south is indeed reasonably reproduced in SST_Assim run, while in AMIP run, it is almost absent. From 2002, in the observation, the negative precipitation anomaly in the north shifted southward and it shows a north-wetter-south-drier pattern again, similar to the first period. SST_Assim shows similar precipitation evolution to observation, except that the south China gets drier earlier than observation and the positive precipitation anomaly in the north extended too far to the south than observation. AMIP run has larger bias in this period, which shows positive precipitation anomaly before 2004 in both north and south, while after 2004 both north and south becomes drier.

Then the spatial patterns of the anomalous precipitation in the three separate decadal periods are shown in both observation and simulation (Fig. 1a–c). It seems that AMIP run performs better than SST_Assim. The Pattern Correlation Coefficient (PCC) of the AMIP (SST_Assim) run with observation is 0.51 (0.29) for the first decadal period. The higher PCC in AMIP run may be resulted from that AMIP is more reasonable in reproducing the anomalous zonal rain belt. It can be seen that in SST_Assim run the precipitation anomaly in the south shows an east-west dipole pattern, different from observation in which the precipitation anomaly is zonally-oriented. In AMIP run the zonal structure of the precipitation anomaly is similar to observation, more reasonable than SST_Assim. It is unclear why SST_Assim run suffers from this bias in simulating the precipitation pattern in East China. Further investigation is required to examine how coupling process and SST assimilation can influence the precipitation pattern in the model.

**Figure 2:**
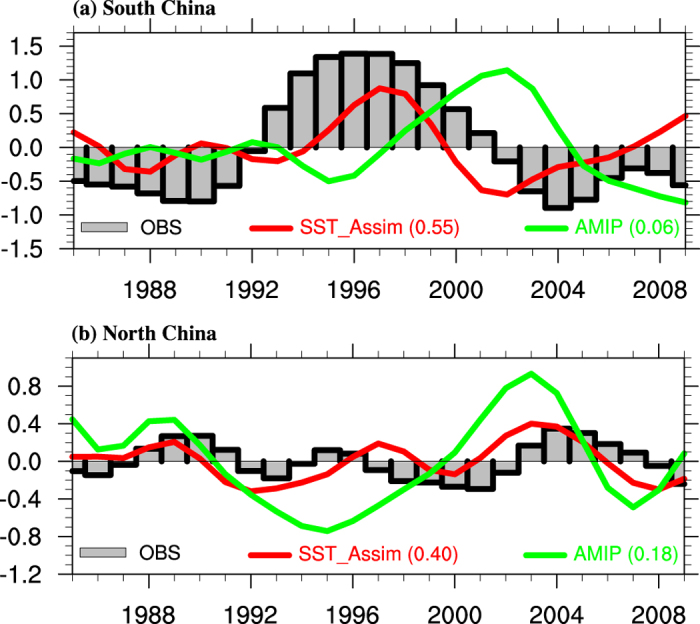
Time evolutions of precipitation anomaly. The time series of precipitation anomaly in the (**a**) south (23°–31°N) and (**b**) north (31°–38°N) part of East China, respectively. Grey bar: observation, red line: SST_Assim, green line: AMIP. The correlation coefficients of SST_Assim and AMIP with observation are also shown. The rainfall anomalies are calculated from removing the climatology of 1985 to 2004 and its long term trend. Maps were generated using NCAR Command Language (The NCAR Command Language (Version 6.1.2) [Software]. (2013). Boulder, Colorado: UCAR/NCAR/CISL/TDD. http://www.ncl.ucar.edu/).

Because the precipitation anomaly in East China usually shows a south-north dipole pattern, separated by 31°N from our above analysis, we further provide the time series of precipitation anomaly in north (31°–38°N) and south (23°–31°N) part of East China, respectively (Fig. 2). Although the precipitation simulation in both AMIP and SST_Assim run has large bias compared to observation, it seems that SST_Assim performs more reasonable than AMIP. For example, SST_Assim run can capture the positive precipitation anomaly in the south from 1994 to1999 and the drier trend after 2000 (Fig. 2a). With respect to the north (Fig. 2b), the magnitude of the precipitation anomaly is more reasonable in SST_Assim run than AMIP. Besides, the positive (negative) precipitation anomaly around 1989 and 2004 (1993 and 2000) can all be captured by SST_Assim run. The correlation coefficients of SST_Assim time series with observation (north/south: 0.40/0.55) is statistically significant at 95% confidence level, higher than AMIP (north/south: 0.18/0.06).

It should be acknowledged that the model indeed has difficulty in simulating the EASM-related precipitation. The same issue is suffered by almost all state-of-the-art climate models. The reason for simulated precipitation bias may be resulted from many factors (e.g., convection parameterization) which cannot be improved through assimilating the SST. We further examined the results of all forcing run and AMIP run in 9 latest CMIP5 models (Methods; Supplementary Table 1). Neither atmosphere-alone model integrations nor coupled climate model runs in CMIP5 captured the decadal variations of EASM circulation (Supplementary Fig. 1 and Fig. 2). It is worth to note that one realization may not enough for the coupled climate model. Using 35 realizations, previous study confirmed that external forcing plays a weakening role on EASM^21^.

**Figure 3:**
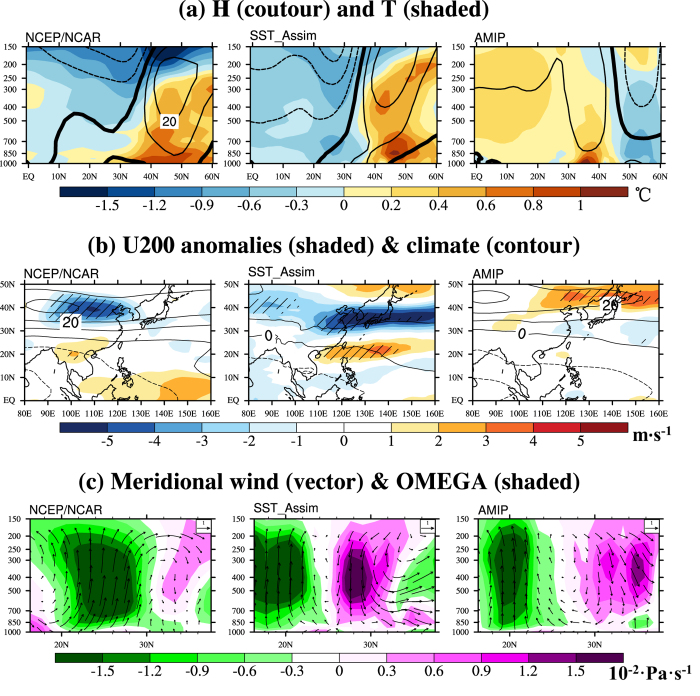
Three-dimensional structure of decadal variation in the early 1990s (1993–2002 minus 1985–1992). The reanalysis (NCEP/NCAR) and simulated (SST_Assim and AMIP) (**a**) zonal–height cross sections in the geopotential height (contour, units: gpm) and temperature (shaded, units: °C), (**b**) the climatological (contour) and anomalous (shaded) zonal wind at 200 hPa (units: m s^−1^), and (**c**) zonal-height cross section in vertical velocity (shaded, units: 10^−2^ Pa s^−1^) and meridional wind (vectors, units: m s^−1^). They are all averaged over 110°–120°E. Maps were generated using NCAR Command Language (The NCAR Command Language (Version 6.1.2) [Software]. (2013). Boulder, Colorado: UCAR/NCAR/CISL/TDD. http://www.ncl.ucar.edu/).

The decadal transition of rain belt in the 1990s is closely associated with the three-dimensional monsoon circulation variation, e.g., WPSH, upper-level westerly jet and tropospheric temperature variations. Figure 3 shows the decadal variation of the three-dimensional structure of the EASM in the 1990s. In East Asia (110°–120°E), the upper level (500–200 hPa) tropospheric temperature exhibits a “south-cool-north-warm” pattern anomaly, with the cooling center located in around 20°–40°N (Fig. 3a). According to the geostrophic balance relation, this cooling may result in the weakening of both the westerly at 40°N and easterly at 20°N (Fig. 3b). The weakened westerly zonal wind crossing 110°E along 30°–40°N indicates a stronger upper-level zonal convergence there, favoring anomalous descending and leading to the reduced summer rainfall in the Yangtze and Huaihe River Valley. According to the “south-negative-north-positive” anomaly of geopotential height and weaker easterly in 20°–30°N (Fig. 3a-b), the WPSH exhibits decadal eastward retreat and northward shift in the 1990s. The anomalous circulation pattern benefits the northwestward transport of the warmer tropical water vapor from the tropical ocean to South China along the southwestern flank of the western North Pacific (WNP), resulting the anomalous ascending in 20°–30°N and positive rainfall. The decadal shifts of the three-dimensional spatial structure in the 1990s are well simulated by SST_Assim, including the eastward retreat and northward shift of the WPSH, and the “south-cool-north-warm” pattern of the upper-level tropospheric temperature, while it is badly simulated by AMIP (Fig. 3). SST_Assim also shows some uncertainties in reflecting the three-dimensional EASM (Fig. 3c). Although the vertical motion (denoted by *ω*) is well simulated by SST_Assim, the meridional circulation is badly reproduced, even worse than AMIP (Fig. 3c).

**Figure 4:**
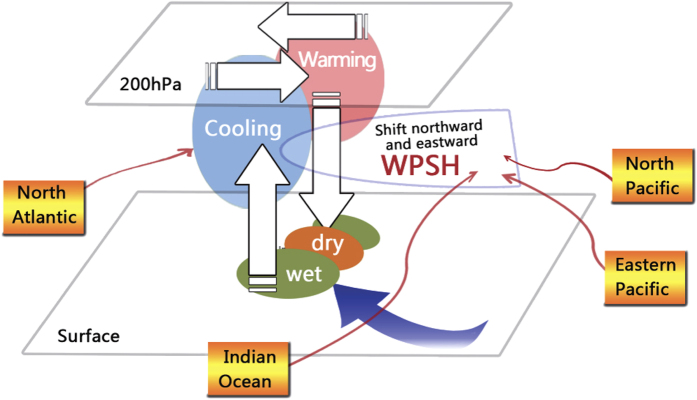
Schematic diagram of the three-dimensional EASM decadal variation in the early 1990s. The vectors in 200 hPa denote the change of upper-level jet. The vertical vectors denote the change of vertical motion. The ellipse between surface and 200 hPa denotes the Western Pacific Subtropical High (WPSH) in 500 hPa. The blue vector in surface denotes the anomalous southeasterly wind associated with the WPSH shift, bringing warm and moist wind from Tropical Ocean to East Asia. The colored circles between surface and 200 hPa denote the shift in troposphere temperature. The colored circles in surface denote the decadal shift of rainfall. This Map was generated using Photoshop.

The schematic diagram of the three-dimensional structure of the decadal variation in the 1990s is shown in Fig. 4. The rain belt transition is associated with the decadal shift of circulation. The successful simulation of the rainfall decadal shift is based on the successful simulation of the three-dimensional spatial structure of the decadal variation of EASM circulation. Several factors are responsible for the decadal variation of EASM and the associated rainfall, including the decadal modulation of EI Niño-Southern Oscillation (ENSO)^25^, Tropical Indian Ocean warming^4^, PDO phase transition^12^, SST variation in the Atlantic^26^, etc. It shall be noted that these signals are all from the ocean, and some of them can be regarded as the internal variability of the climate system. Thus, our successful simulation sits on the fact that the “true” evolution of the SST is assimilated into the coupled model. Separating the respective contribution from each factor mentioned above may need more numerical experiments and we will further investigate into this issue in the future.

## Discussion

Three factors stand out as to why the decadal variation of the EASM can be captured in our study. First of all, our system maintains adequate air-sea interaction as a fully coupled model, because the atmospheric feedback plays a major role in determining local SST over the Asian-Pacific summer monsoon regions^27^.

**Figure 5:**
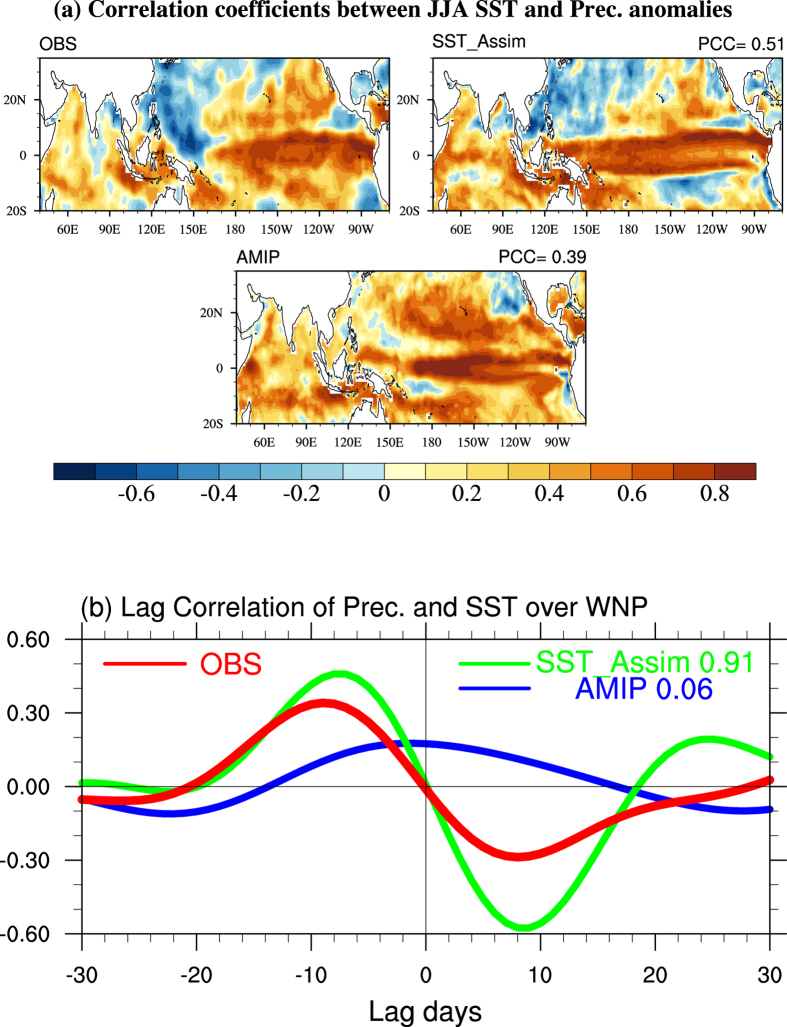
Relationship between SST and precipitation. (**a**) Correlation coefficients between the June-August averaged SST and precipitation anomalies derived from GPCP and Hadley Centre Sea Ice and Sea Surface Temperature dataset (HadISST), SST_Assim and AMIP. The PCCs mean the pattern correlation coefficients between the simulation (SST_Assim and AMIP) and the observation. (**b**) The lead-lag correlations of intraseasonal rainfall and local SST for the observations (daily GPCP precipitation and daily AVHRR SST), SST_Assim and AMIP. The values in (**b**) denote the correlation coefficients between the simulation (SST_Assim and AMIP) and the observation. Daily rainfall and SST data are filtered with only 20–100-day variability retained. Maps were generated using NCAR Command Language (The NCAR Command Language (Version 6.1.2) [Software]. (2013). Boulder, Colorado: UCAR/NCAR/CISL/TDD. http://www.ncl.ucar.edu/).

Even the dynamical core and physical parameterization schemes in AGCMs have been significantly improved^28^, the latest AMIP simulations show large uncertainty in simulating EASM decadal variations (Supplementary Fig. 1). The observed negative relationship between local SST and precipitation anomalies in WNP region are successfully reflected by SST_Assim, with pattern correlation coefficient (PCC) between SST_Assim and the observation is 0.51; while it is not the case in AMIP, with PCC is 0.39 (Fig. 5a). The negative feedback of this mechanism is that: high (low) SST tends to induce (suppress) convection and large precipitation; and induced (suppressed) convection tends to decrease (increase) the SST because it cuts (increases) the short wave heating^27^. On intraseasonal time scale, the positive (negative) SST leads to positive (negative) precipitation in about 10 days in the SST_Assim and observation. Meanwhile, when positive (negative) precipitation happens, it will lead to negative (positive) SST. This actually reflects a negative feedback of rainfall-SST. The correlation coefficient between SST_Assim and the observation is 0.91. In AMIP, positive SST is almost in phase with rainfall, with the correlation coefficient between AMIP and the observation is only 0.06 (Fig. 5b). AMIP’s failure can be attributed to lacking of atmospheric feedback to the ocean in the experiment design^27^. In AGCMs alone simulation, the air-sea interaction cannot be correctly represented, contributing to deficiencies in rainfall simulations over East China^21^. Comparing the AMIP and coupled model simulations from CMIP5, previous study found that the EASM was better simulated in the coupled model than in the AGCMs^29^. Simple model studies also demonstrated that specification of historical SST anomalies as a boundary condition cannot generally lead to a correct simulation of low-frequency atmospheric variance^30^.

Another substantial advantage of our system is that the oceanic fields will not deviate far from the observations through ocean data assimilation. Although only SST field is assimilated, the oceanic fields, i.e. sea surface height, temperature, salinity, zonal and meridional current, will adjust dynamically based on background error covariance (Methods). Through data assimilation techniques, our system can input the signal of internal variability in the model. Conventionally, it is impossible for a CGCM to reproduce the actual climate fluctuations without inputting observed information. Bad simulations of SST decadal oscillations (e.g., PDO index, Supplementary Fig. 3) may fail the EASM simulation. The main deficiency of all forcing run showed by Supplementary Fig. 2 may be that using external forcing cannot capture the observed SST oscillations. It is worth noting that the observed SST includes both signals from external forcing and internal variability. For example, it is confirmed that the tropical Indian Ocean SST warming during the past half century is dominated by external forces, especially GHGs^31^,^32^,^33^. Since all forcing runs fail to simulate the decadal shifts of EASM, our experiments imply that the internal variability plays a dominant role in EASM decadal transitions.

The assimilation interval adopted in our experiment (every 7 days) is the third factor that counts. Due to the fact that the random synoptic process of atmosphere, on its own, cannot last for more than two weeks^34^, we conducted four SST_Assim experiments with 1 day, 3 days, 7 days and 14 days assimilation interval respectively. The 1 day and 3 days assimilation show similar results with AMIP, in which the local summer SST and rainfall are almost positively correlated in WNP (Supplementary Fig. 4), while 7 days and 14 days assimilation run reproduce the observed negatively correlation. The intensity and variance magnitude of precipitation in 1 day and 3 days assimilation are much lower than the observation, while 7 days assimilation shows the best results (Supplementary Table 2 and Fig. 5). A shorter time interval less than 7 days may interrupt the atmospheric feedback process and destroy the persistence of atmospheric disturbance. A time interval longer than 7 days will constrain the ocean fields with fewer observations. Therefore, we believe that the 7 days’ time interval is suitable in our simulation.

## Methods

### Model

The model used in this study is a fully coupled climate system model (CAS-ESM-C) developed in the Institute of Atmospheric Physics (IAP), Chinese Academy of Science (CAS)^22^, which consists of an atmospheric component (IAP AGCM4)^35^, an oceanic component (LICOM1.0)^36^, a land component (Community Land Model, CLM3) and a sea ice component (Community Sea Ice Model, CSIM5)^37^. A coupler (cpl6), developed at the National Center for Atmospheric Research (NCAR), is employed in this model to couple each component model together^22^. The performances of this model in some basic aspects have been systematically evaluated^38^,^39^,^40^,^41^.

### Experiments

Two types of numerical experiments are designed as follow: 1) SST_Assim: The observed SST is assimilated into the ocean component of CAS-ESM-C once every 7 days during the free coupled integration. Coupling is performed without any adjustment of SST and fluxes. 2) AMIP: The atmospheric component is forced with observed monthly SST as the oceanic boundary condition. The gridded daily SST data used for assimilation are infrared satellite data from Advanced Very High Resolution Radiometer (AVHRR)^24^, spanning from 1981 to 2014. The data assimilation scheme is Ensemble Optimal Interpolation (EnOI)^42^,^43^.

### Datasets

Four sets of observational monthly data are used: 1) a high resolution gridded precipitation data across China produced by National Climate Centre China of China Meteorological Administration^44^. This dataset is derived from the interpolation from over 2,400 observing stations in China and available in 0.5° × 0.5° spatial resolution from 1951 to 2012. 2) Global Precipitation Climatology Project (GPCP) data^45^,^46^, they are available in both daily and monthly with 1° × 1° and 2.5° × 2.5° resolutions, respectively; 3) Hadley Centre Sea Ice and Sea Surface Temperature dataset (HadISST)^47^; 4) NCEP/NCAR reanalysis datasets^48^, the low-level meridional wind component (V850), vertical velocity (−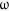), geopotential height (H), air temperature (T) and upper-level zonal wind (U200) are used in this study.

The outputs of AMIP and all forcing runs from 9 CMIP5 models are analyzed in this study (see Supplementary Table 1 for model descriptions). Only the first realization of all models, during 1979–2008 for AMIP and 1961–2005 for historical experiment, are analyzed in this study. The all forcing runs in CMIP5 are the coupled historical simulations which are forced by external forcings (e.g. GHGs, anthropogenic aerosols, solar variability and volcanic eruptions)^49^.

### Assimilation scheme (EnOI)

The following equations are employed to compute the analysis based on the background field:


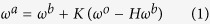



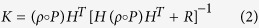



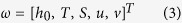



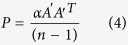


The subscripts a, b and o denote the analysis, background and observation, respectively; And the superscript T denotes matrixes transpose; *ω* means the state vector including sea surface height (*h*_0_), temperature (T), salinity (S), zonal and meridional current (u and v); R denotes the observation error covariance matrix; H is the observation operator that interpolates from the model space to observation space; 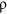 denotes a localizing correlation function used to remove the effects of sampling error due to the smaller ensemble size than the dimension of the model space^50^. The circle between 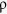 and P denotes a Schur product; P means the background error covariance matrix, which can be calculated from a matrix *A*′, consisting of n ensemble members which are taken from the model state anomalies. The stationary ensemble of model states adopted in EnOI is usually sampled during a long-term control run to estimate the structure of the background error covariance^42^. Thus before our assimilation experiment, a 200-year control integration is conducted in advance to derive the stationary ensemble of model states. The stationary ensemble size n equals 108 in this study. Furthermore, the ensembles used in the assimilation are dependent on different months, in order to adequately describe the distinct characteristics of the oceanic current in different months^51^.

In this study, the EnOI scheme is adopted to assimilate observed SST data into the ocean component of CAS-ESM-C. Actually, the same assimilation scheme has been adopted in establishing the global ocean data assimilation system (ZFL_GODAS)^52^ and the ocean data assimilation system in the Indian Ocean and west Pacific Ocean^51^.

## Additional Information

**How to cite this article**: Lin, R. *et al*. Decadal shifts of East Asian summer monsoon in a climate model free of explicit GHGs and aerosols. *Sci. Rep.* **6**, 38546; doi: 10.1038/srep38546 (2016).

**Publisher's note:** Springer Nature remains neutral with regard to jurisdictional claims in published maps and institutional affiliations.

## Supplementary Material

Supplementary Information
