# Supplementary material for: Decadal shifts of East Asian summer monsoon in a climate model free of explicit GHGs and aerosols
Source: Sci Rep. 2016 Dec 9;6:38546. doi: 10.1038/srep38546 (PMC5146936; doi:10.1038/srep38546)
Supplement: Supplementary Information [file srep38546-s1.pdf]

Decadal shifts of East Asian summer monsoon in a climate model free of explicit GHGs and aerosols

Renping Lin, Jiang Zhu* and Fei Zheng

International Center for Climate and Environment Sciences, Institute of Atmospheric Physics, Chinese Academy of Sciences, Beijing 100029

Correspondence to Jiang Zhu (email: jzhu@mail.iap.ac.cn)

Supplementary Table 1 Institution, model designation, and horizontal and vertical resolution of the CMIP5 models used in this study.

Institution	Model designation	AGCM horizontal/vertical resolution	OGCM horizontal/vertical resolution
National Science Foundation, Department of Energy, National Center for Atmospheric Research	CESM1-CAM5	1.25 °lon × 0.9 °lat L26	1.1 °lon × 0.27 °-0.54 ° lat L60
Commonwealth Scientific and Industrial Research Organization in collaboration with Queensland Climate Change Centre of Excellence	CSIRO-Mk3.6.0	T63 L18	1.875 °lon × ~0.9375 ° lat L31
LASG, Institute of Atmospheric Physics, Chinese Academy of Sciences and CESS,Tsinghua University	FGOALS-g2	128 × 60 L26	360 × 196 L30
NOAA Geophysical Fluid Dynamics Laboratory	GFDL-ESM2M<br>GFDL-CM3	M45 L24	360 × 200 L50
NASA Goddard Institute for Space Studies	GISS-E2-R	2.5 °lon × 2 °lat L40	1.25 °lon × 1 °lat L32
Met Office Hadley Centre	HadGEM2-CC<br>HadGEM2-A	N96 L60	1 °lon × 0.3 °-1.0 °lat L40
Institut Pierre-Simon Laplace	IPSL-CM5A-LR	96 × 95 L39	2 °lon × 2 °lat L31
Max Planck Institute for Meteorology	MPI-ESM-LR	T63 L47	GR15 L40
Meteorological Research Institute	MRI-CGCM3	TL159 L48	1 °lon × 0.5 °lat L51

Supplementary Table 2 The climatological mean and standard deviation of area-averaged precipitation time series in the area east of Philippines, which are shown in Supplementary Fig. 6. These results are derived from the observation and four SST_Assim simulations (1-day, 3-day, 7-day and 14-day). The "correlation coefficients with OBS" denote the correlation coefficients between simulated and observed time series which are shown in Supplementary Fig. 6.

	**OBS**	**1-day**	**3-day**	**7-day**	**14-day**
climate mean (mm day^{-1})	5.38	2.63	3.06	5.09	5.47
standard deviation (mm day^{-1})	2.59	1.36	1.76	3.11	3.22
correlation coefficients with OBS	-	0.54	0.45	0.70	0.65

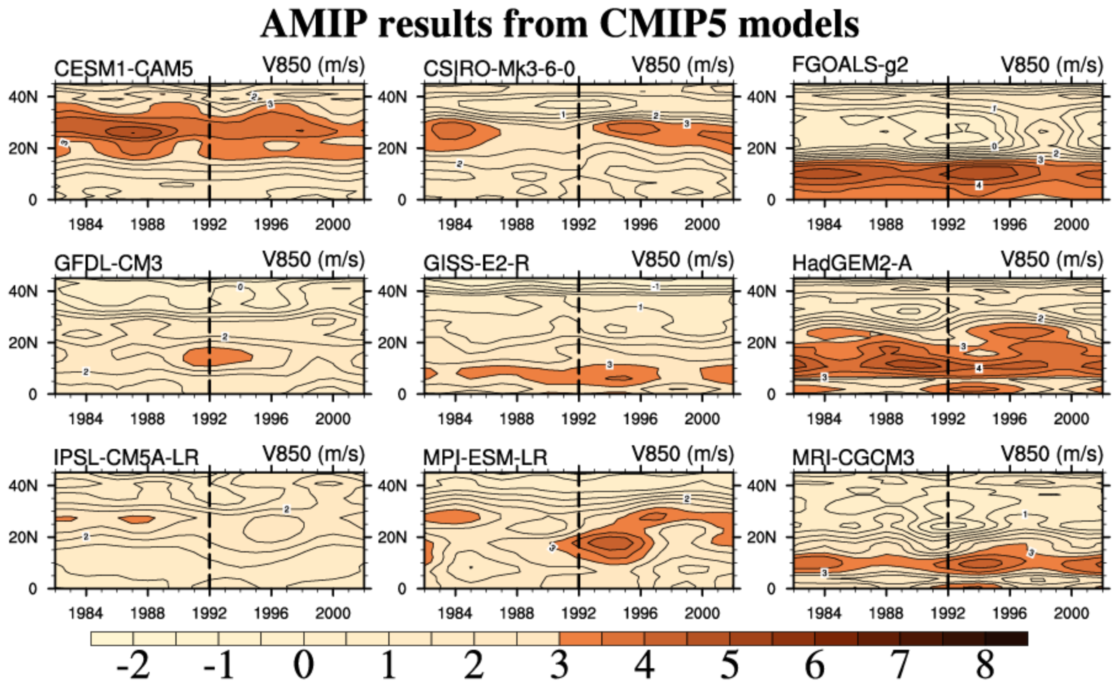

Supplementary Figure 1 Decadal Variation of low level south wind simulated by AMIP-type simulation of the CMIP5 models. The time-latitude distribution of meridional 850-hPa wind over East China (110-120 °E) derived from AMIP run results from the 9 CMIP5 models. These models are descripted in Supplementary Table 1. Maps were generated using NCAR Command Language (The NCAR Command Language (Version 6.1.2) [Software]. (2013). Boulder, Colorado: UCAR/NCAR/CISL/TDD. http://www.ncl.ucar.edu/).

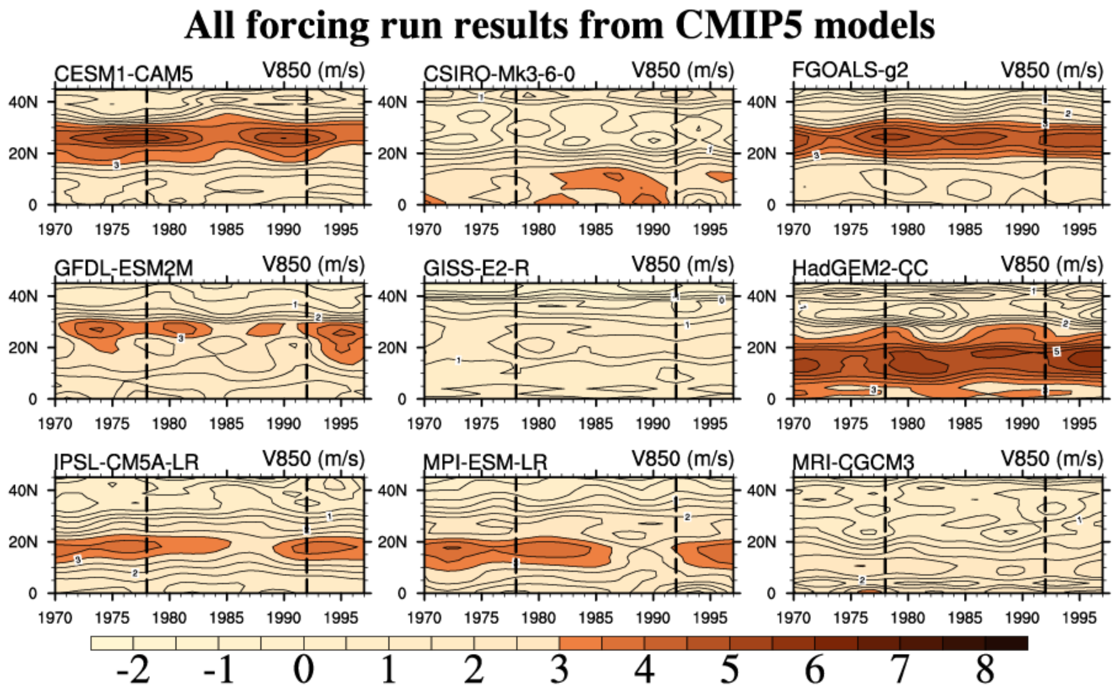

Supplementary Figure 2 Decadal Variation of low level south wind simulated by historical run of the CMIP5 models. The time-latitude distribution of meridional 850-hPa wind over East China (110-120 °E) derived from historical run results from the 9 CMIP5 models. These models are descripted in Supplementary Table 1. Maps were generated using NCAR Command Language (The NCAR Command Language (Version 6.1.2) [Software]. (2013). Boulder, Colorado: UCAR/NCAR/CISL/TDD. http://www.ncl.ucar.edu/).

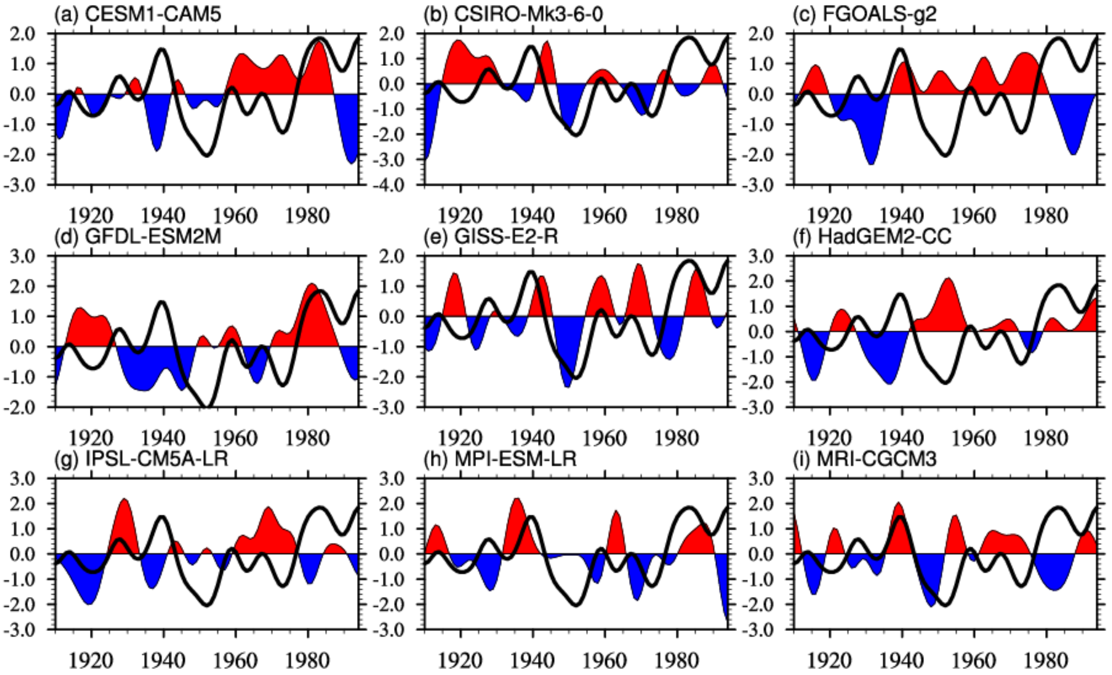

Supplementary Figure 3 Time series of Pacific Decadal Oscillation (PDO) index during 1900-2005 simulated by 9 CMIP5 models. The time series of Pacific Decadal Oscillation (PDO) yearly index during 1900-2005 derived from the same 9 CMIP5 model. The black lines indicate the observation. Maps were generated using NCAR Command Language (The NCAR Command Language (Version 6.1.2) [Software]. (2013). Boulder, Colorado: UCAR/NCAR/CISL/TDD. http://www.ncl.ucar.edu/).

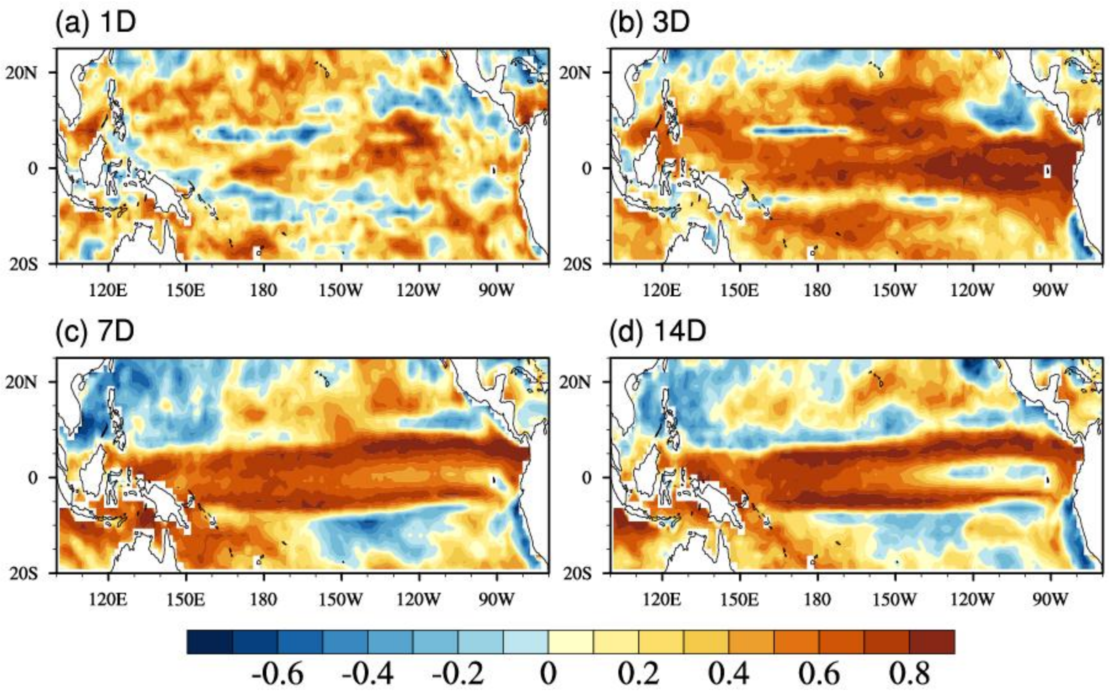

Supplementary Figure 4 Spatial Pattern of correlation coefficients between the June-August SST and precipitation anomalies in different assimilation time interval experiments. Correlation coefficients between the June-August SST and precipitation anomalies derived from (a) 1 day (1D) assimilation run, (b) 3 days (3D) assimilation run, (c) 7 days (7D) assimilation run and (d) 14 days (14D) assimilation run. Maps were generated using NCAR Command Language (The NCAR Command Language (Version 6.1.2) [Software]. (2013). Boulder, Colorado: UCAR/NCAR/CISL/TDD. http://www.ncl.ucar.edu/).

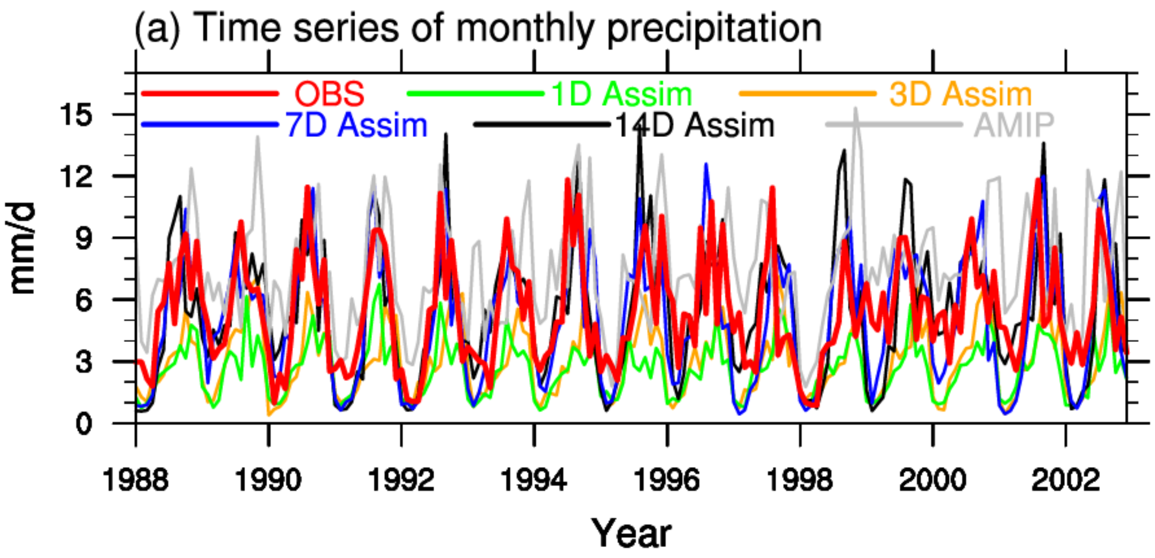

Supplementary Figure 5 Time series of precipitation (mm/d) in the area east of the Philippines (5–25 °N, 125–150 °E) in observation and different assimilation time interval experiments. Time series of precipitation (mm/d) in the area east of the Philippines (5–25 °N, 125–150 °E) for observation (red line), AMIP run (gray line), 1-day (1D) Assim run (green line), 3D Assim run (orange line), 7D Assim run (blue line) and 14D Assim run (black line). The observed monthly data is GPCP monthly data with 2.5 °×2.5 ° resolution. Maps were generated using NCAR Command Language (The NCAR Command Language (Version 6.1.2) [Software]. (2013). Boulder, Colorado: UCAR/NCAR/CISL/TDD. http://www.ncl.ucar.edu/).
